# HEALTH AND WELL-BEING ACROSS ECOLOGICAL SYSTEMS AND TIME IN MID LIFE AND LATE ADULTHOOD

**DOI:** 10.1093/geroni/igad104.1762

**Published:** 2023-12-21

**Authors:** Kelly Cichy, Robert Stawski, Robert Stawski

## Abstract

Health and well-being are shaped by peoples’ experiences and ecological contexts (Bronfenbrenner, 2005), reflecting the influence of complex person-context relationships. For example, experiences (e.g., daily stressors, early childhood adversity) occur within microlevel (e.g., marital relationships, neighborhoods) and macrolevel (e.g., societal inequalities) contexts, impacting both daily well-being and healthy aging. Further, a robust literature highlights the significance of naturally occurring everyday experiences for a range of health outcomes, underscoring how daily experiences have immediate and lasting effects on individual physical and cognitive health and psychological well-being. Drawing on Bronfenbrenner’s Ecological Model, the current symposium features novel research examining health and well-being outcomes across diverse ecological systems and time scales across midlife and old age, with the goal of articulating how innovative approaches for studying person-context interactions can be leveraged to understand both proximal and long-term health and well-being. Polenick and colleagues will examine associations between daily negative and positive events and well-being among heterosexual married couples living with early-stage dementia. Cichy et al. will examine gender differences in the prospective effects of daily stress processes on long-term chronic disease risk in midlife and later life. Next, Turner and Mogle will examine how positive and negative affect mediate the associations between daily memory lapses and psychological well-being. Finally, Choi, Munoz, Scott, and Sliwinski will examine whether current positive (i.e., neighborhood cohesion) and negative (i.e., neighborhood violence) neighborhood contexts moderate the effect of childhood stress exposure on cognitive function in adulthood. Robert Stawski will synthesize the presentations and offer insights for future research.

